# Modulating design parameters to drive cell invasion into hydrogels for osteochondral tissue formation

**DOI:** 10.1016/j.jot.2023.07.001

**Published:** 2023-09-04

**Authors:** Andrea Schwab, Marinus A. Wesdorp, Jietao Xu, Florencia Abinzano, Claudia Loebel, Marc Falandt, Riccardo Levato, David Eglin, Roberto Narcisi, Martin J. Stoddart, Jos Malda, Jason A. Burdick, Matteo D'Este, Gerjo J.V.M. van Osch

**Affiliations:** a Department of Orthopaedics and Sports Medicine, Erasmus MC, University Medical Center Rotterdam, the Netherlands; b AO Research Institute Davos, AO Foundation, Davos Platz, Switzerland; c Department of Orthopedics, University Medical Center Utrecht, Utrecht, the Netherlands; d Department of Bioengineering, University of Pennsylvania, Philadelphia, PA, USA; e Department of Clinical Sciences, Faculty of Veterinary Sciences, Utrecht University, Utrecht, the Netherlands; f Mines Saint-Etienne, University Jean Monnet, INSERM, UMR 1059, Saint-Etienne, France; g Advanced Organ Bioengineering and Therapeutics, Faculty of Science and Technology, TechMed Center, University of Twente, Enschede, the Netherlands; h Department of Otorhinolaryngology, Erasmus MC, University Medical Center Rotterdam, Rotterdam, the Netherlands; i Department of Biomechanical Engineering, Faculty of Mechanical, Maritime, and Materials Engineering, Delft University of Technology, Delft, the Netherlands

**Keywords:** Cartilage, Cells, Hydrogels, Regenerative medicine, Tissue engineering

## Abstract

**Background:**

The use of acellular hydrogels to repair osteochondral defects requires cells to first invade the biomaterial and then to deposit extracellular matrix for tissue regeneration. Due to the diverse physicochemical properties of engineered hydrogels, the specific properties that allow or even improve the behaviour of cells are not yet clear. The aim of this study was to investigate the influence of various physicochemical properties of hydrogels on cell migration and related tissue formation using *in vitro*, *ex vivo* and *in vivo* models.

**Methods:**

Three hydrogel platforms were used in the study: Gelatine methacryloyl (GelMA) (5% wt), norbornene hyaluronic acid (norHA) (2% wt) and tyramine functionalised hyaluronic acid (THA) (2.5% wt). GelMA was modified to vary the degree of functionalisation (DoF 50% and 80%), norHA was used with varied degradability via a matrix metalloproteinase (MMP) degradable crosslinker and THA was used with the addition of collagen fibrils. The migration of human mesenchymal stromal cells (hMSC) in hydrogels was studied *in vitro* using a 3D spheroid migration assay over 48h. In addition, chondrocyte migration within and around hydrogels was investigated in an *ex vivo* bovine cartilage ring model (three weeks). Finally, tissue repair within osteochondral defects was studied in a semi-orthotopic *in vivo* mouse model (six weeks).

**Results:**

A lower DoF of GelMA did not affect cell migration *in vitro* (p ​= ​0.390) and led to a higher migration score *ex vivo* (p ​< ​0.001). The introduction of a MMP degradable crosslinker in norHA hydrogels did not improve cell infiltration *in vitro* or *in vivo.* The addition of collagen to THA resulted in greater hMSC migration *in vitro* (p ​= ​0.031) and *ex vivo* (p ​< ​0.001). Hydrogels that exhibited more cell migration *in vitro* or *ex vivo* also showed more tissue formation in the osteochondral defects *in vivo,* except for the norHA group. Whereas norHA with a degradable crosslinker did not improve cell migration *in vitro* or *ex vivo*, it did significantly increase tissue formation *in vivo* compared to the non-degradable crosslinker (p ​< ​0.001).

**Conclusion:**

The modification of hydrogels by adapting DoF, use of a degradable crosslinker or including fibrillar collagen can control and improve cell migration and tissue formation for osteochondral defect repair. This study also emphasizes the importance of performing both *in vitro* and *in vivo* testing of biomaterials, as, depending on the material, the results might be affected by the model used.

The translational potential of this article: This article highlights the potential of using acellular hydrogels to repair osteochondral defects, which are common injuries in orthopaedics. The study provides a deeper understanding of how to modify the properties of hydrogels to control cell migration and tissue formation for osteochondral defect repair. The results of this article also highlight that the choice of the used laboratory model can affect the outcome. Testing hydrogels in different models is thus advised for successful translation of laboratory results to the clinical application.

## Graphical abstract

**Figure:**
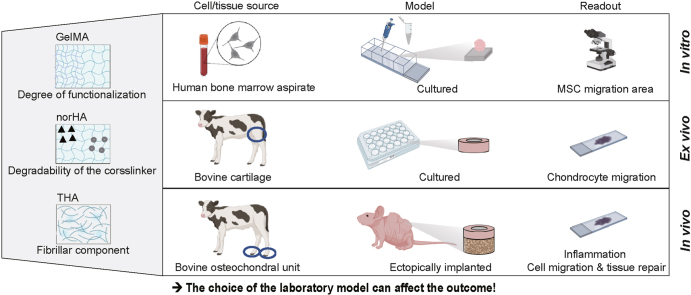

## 1 Introduction

Current matrix-based approaches for the repair of (osteo-) chondral defects, like autologous matrix-induced chondrogenesis (AMIC, a combination of microfracture with a collagen scaffold) and matrix-assisted chondrocyte implantation (MACI), have improved clinical outcomes and lowered revision rates when compared to microfracture alone [[1], [2], [3]]. Although these treatments lead to improved function and reduced pain, they fail to repair defects fully functional for the long-term [4]. Acellular biomaterial-assisted approaches are an attractive alternative to cell-based procedures for the treatment of small focal defects, particularly due to the elimination of donor-site morbidity and the possibility of a single-stage procedure [5]. Importantly, it has been shown that biomaterials help to improve the preservation of the cartilage tissue surrounding the defect [6]. However, to repair cartilage fully, improved mobilisation and infiltration of cells residing in the knee into the biomaterials are needed [[7], [8], [9]].

Injectable hydrogels are one promising group of biomaterials to fill complex defects of any shape and location through a minimally invasive approach [10,11]. Despite the extensive research performed *in vitro* on the chondro-inductive properties of hydrogels, these biomaterials often fail upon implantation *in vivo.* Failure is related to the challenge of retaining the material in the defect area, which limits cell invasion [12]. Integration of the biomaterial with the tissues adjacent to the defect, results mainly from the infiltration of host cells, followed by matrix deposition [13]. Thus, rapid cell infiltration is an important step in an integrative defect repair strategy [14]. Cell infiltration is not only relevant for acellular hydrogel-assisted repair, but also of interest for approaches where cells are encapsulated within a hydrogel to integrate the repair tissue into the host tissue [15]. This highlights the need to understand how physicochemical properties of hydrogels influence cell migration and which modifications may improve cell migration from surrounding tissues for osteochondral defect repair.

Since collagen and glycosaminoglycan are major components in the ECM of connective tissue, collagen and hyaluronic acid (HA) based hydrogels have attracted interest in cartilage tissue engineering approaches [16]. Gelatine, the product of denatured collagen, modified with methacryloyl groups (GelMA), is an emerging and widely used hydrogel that exhibits tunable material properties while maintaining regions with cell adhesives (e.g. arginine-glycine-aspartate (RGD)) and degradable sequences [17,18]. The crosslinking density in GelMA hydrogels is controlled by the degree of functionalisation (DoF), which is the extent of functionalisation with methacryloyl groups that alters crosslinking. GelMA at a fixed concentration, but an increase in DoF leads to a higher crosslinking density and thus a smaller mesh size [19]. More recently, a lower degree of functionalization of the GelMA50 has been associated with a faster enzymatic degradation kinetic [20]. For endothelial cells it has been shown that a lower DoF supports greater endothelial cell derived capillary-like-network formation [21]. Whether this hydrogel modification also improves cell migration in the osteochondral environment and supports tissue repair has not yet been studied.

The most widely used proteoglycan hydrogels in biomedicine are HA and its derivatives. HA-based biomaterials are often functionalised with biochemical cues (e.g.*,* chondroinductive or chondroconductive peptides, the addition of fibrous components) and/or biophysical cues (mechanical properties, mesh size, porosity) to stimulate (endogenous) cell infiltration and cartilaginous matrix deposition [14,22,23]. To improve the tunability of mechanical properties, the interaction with the host tissue or the rheological properties and printability, functionalization of HA-based hydrogels (e.g. with thiol-norbornene or tyramine) have been introduced [[24], [25], [26]]. Norbornene hyaluronic acid (norHA) is an attractive biomaterial due to its excellent printability, tunable properties and the ability for *in situ* crosslinking [27]. Tyramine functionalized HA (THA) is characterized by its enhanced binding to the cartilage host tissue via the formation of di-tyrosine bonds between THA and cartilage ECM [28]. Together with its tunable properties, THA is also an attractive hydrogel for bioprinting [[29], [30], [31]]. The main limitation of HA-based materials are the limited cell adhesion [32,33]. One approach to overcome this is to combine THA with either RGD, collagen or gelatine to increase cell attachment and cell spreading [30,34,35]. Beyond the possibility of varying RGD concentration, the use of a degradable crosslinker, specifically the use of a matrix metalloproteinase (MMP) cleavable crosslinker, has allowed mesenchymal stromal cells (MSC) spreading and mechano-response when embedded in the hydrogel [36].

The above-introduced hydrogel biomaterials (GelMA, norHA and THA) are attractive for (osteo-) chondral repair due to their chondrogenic potential and printability [[27], [28], [29], [30],^34^,[36], [37], [38], [39], [40], [41]]. Thus, this study aimed to investigate the effect of selected hydrogel modifications on human hMSC and chondrocyte migration *in vitro* and related tissue formation for (osteo-) chondral defect repair *in vivo*. We hypothesised that a lower crosslinking density in GelMA, use of a degradable crosslinker with norHA precursors, as well as the addition of collagen fibrils in THA hydrogels would increase cell migration and improve tissue formation. Cell migration into the hydrogel was investigated using three models: (1) hMSC spheroids seeded within hydrogels for *in vitro* migration, (2) endogenous chondrocyte migration in a cartilage ring model *ex vivo*, and (3) cell migration and tissue formation in a semi-orthotopic mouse model *in vivo*. While the first two models focus on a single cell type, the *in vivo* model was chosen to study the interplay of multiple cell types within the osteochondral defect environment.

## 2 Materials and methods

### 2.1 Biomaterial preparation and characterization

#### 2.1.1 Hydrogel preparation

**Figure 1:**
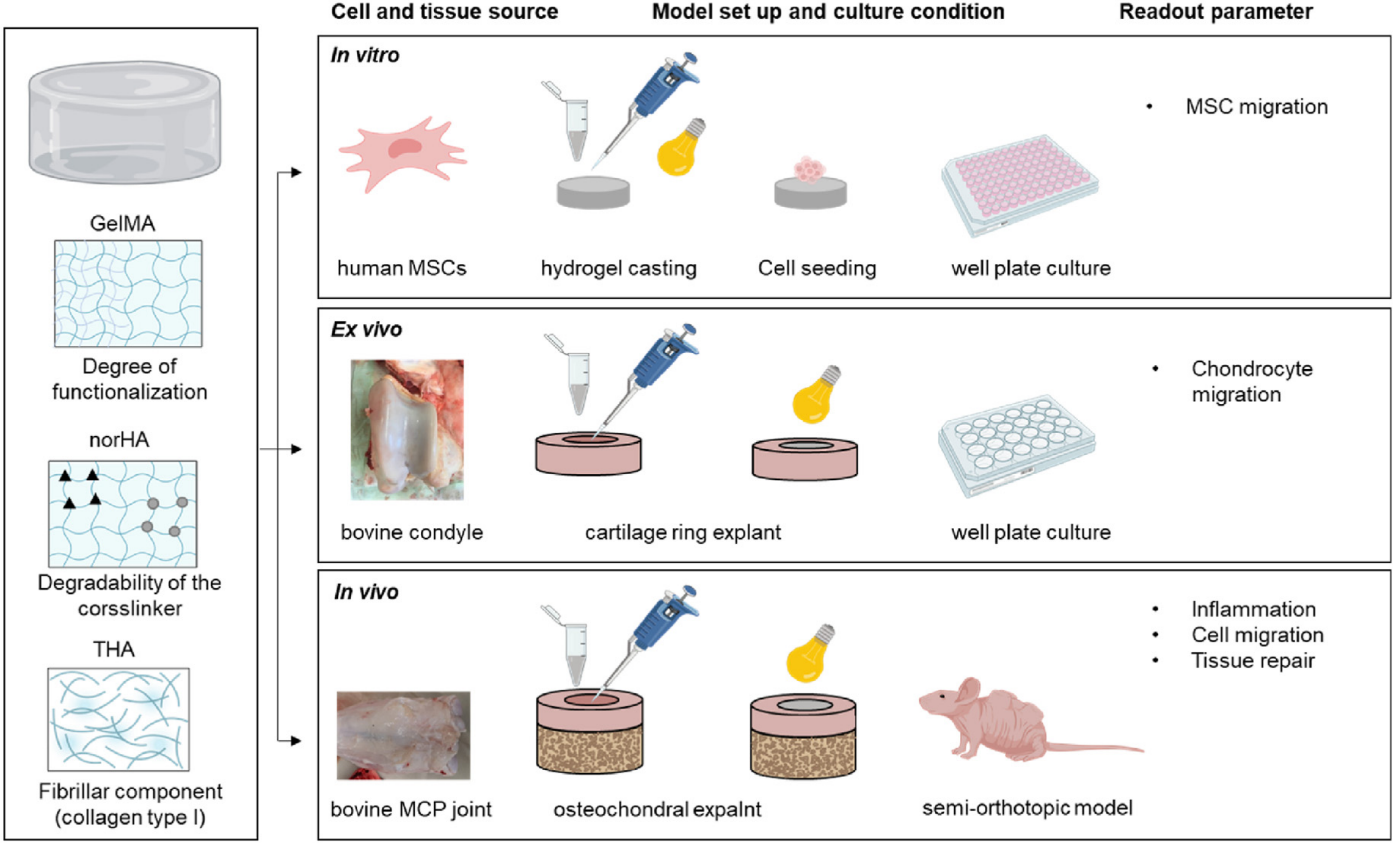
**Graphical illustration of the in *vitro, ex vivo* and *in vivo* models to evaluate acellular hydrogels for (osteo-) chondral repair.** Three hydrogel types were evaluated for cell migration and tissue formation in osteochondral defects. Comparisons included the degree of functionalization (50% and 80%) for gelatine methacryloyl (GelMA hydrogels), the degradability of matrix metalloprotease (MMP) degradable versus non-degradable (1,4-Dithiothreitol, DTT) crosslinkers for norbornene-modified hyaluronic acid (HA) and the addition of fibrillar collagen on tyramine modified HA (THA) hydrogels. Images were created with Biorender.com.

Two HA-based materials (THA, norHA) and a gelatine-based material (GelMA) were evaluated by MSC cell migration *in vitro*, chondrocyte migration from cartilage explants *ex vivo*, cell migration, and osteochondral defect repair in a semi-orthotopic model *in vivo (*Fig. 1*).* For the *in vitro* migration study, hydrogels were crosslinked in an 8-well plate prior to cell seeding. For the *in vivo* and *ex vivo* model, hydrogel precursors were injected in the defects of either cartilage rings or osteochondral explants and photo-crosslinked.

##### 2.1.1.1 Gelatine methacryloyl (GelMA)

GelMA was synthesized with a DoF of 50% (GelMA50) and 80% (GelMA80). Based on the protocol described by Melchels et al., 0.6 ​g methacrylic anhydride (Sigma Aldrich 92%) per g of gelatine (type A from porcine skin, 300 ​g Bloom, Sigma Aldrich) was used (10% gelatine in PBS, 50°C, 1h) for GelMA80. For GelMA50, 0.036 ​g methacrylic anhydride per g of gelatine was used [42,43]. GelMA (50 ​mg/ml) was reconstituted at 60°C before adding the photo crosslinker ruthenium (0.5 ​mM) and sodium persulfate (5.0 ​mM) with subsequent photo-crosslinking (15 ​min, 3 ​cm distance, AVIDE lamp). The variable of the two GelMA formulations used in this study were the DoF (50% or 80%).

##### 2.1.1.2 Norbonene hyaluronic acid (norHA)

NorHA was synthesized as previously described and reconstituted with either a non-degradable 1,4-Dithiothreitol (DTT, 0.22 ​mg/ml, Sigma Aldrich, Saint Louis, USA) crosslinker or a MMP cleavable crosslinker (2.55 ​mg/ml, GCNSVPMS↓MRGGSNCG, Lot: U1432DL070-1/PE2401, GenScript, Piscataway Township, USA) [41,44]. NorHA was reconstituted at a final polymer concentration of 20 ​mg/ml. NorHA precursors (norHA-DTT and norHA-MMP) contained thiolated RGD sequences (GCGYGRGDSPG, 1.0 ​mM, U0140DA260-1, Lot: 94230930001/PE8559, GenScript). Photo-crosslinking (20 ​min, 3 ​cm distance, AVIDE lamp) was achieved using photo-initiators ruthenium and sodium persulfate (Advanced Biomatrix, 5248-1KIT) followed by hydrogel gelation. The choice of the crosslinker (MMP degradable or non-degradable DTT) was the variable to prepare two norHA formulations.

##### 2.1.1.3 Tyramine modified hyaluronic acid (THA)

THA with a degree of substitution of 6% was synthesized as described previously [45]. THA was reconstituted (25 ​mg/ml) and photoinitiator Eosin Y (0.02 ​mg/ml, Sigma Aldrich) was added. THA (25 ​mg/ml final concentration) and THA-collagen (THA 12.5 ​mg/ml and 2.5 ​mg/ml collagen 1 isolated from rat tails, Corning, Bedford, USA) hydrogels were enzymatically crosslinked using peroxidase from horseradish (0.3 U/ml, Sigma Aldrich) and hydrogen peroxide (120 ​ppm, Carl Roth, Karlsruhe, Germany) with subsequent photo-crosslinking (10 ​min, 3 ​cm distance, AVIDE lamp, Well-Com Vertriebs GmbH, Gelsenkirchen, Germany) [34]. The variable for the two THA formulations was the addition of the fibrillary component (THA-col) compared to THA alone.

#### 2.1.2 Nuclear magnetic resonance (NMR) spectrum

The hydrogel precursor materials were characterized by ^1^H NMR spectroscopy to confirm the molecular structure, and to assess purity and degree of functionalization. All materials were dissolved in deuterium oxide (D_2_O).

#### 2.1.3 Dynamic mechanical analysis

Hydrogel mechanical properties were assessed using a dynamic mechanical analyzer (DMA Q800, TA Instruments, The Netherlands). Hydrogels in phosphate buffered saline (PBS, Sigma Aldrich) were analysed in unconfined uniaxial compression test (room temperature, n ​= ​5 samples per condition) to measure their compressive moduli. A preload of 0.01 ​N was applied to the hydrogels, followed by a ramp force of 2 ​N/m until a maximum force of 8 ​N was reached. The compressive modulus (Young's modulus) was calculated as the slope of the linear elastic range of the stress/strain curve. To measure the stress-relaxation response, a constant strain was applied (preload of 0.001 ​N, room temperature, n ​= ​5 samples per condition) to hydrogels. After applying a constant strain of 20% for 2 ​min, the hydrogel response was measured over a period of 1 ​min. The relaxation of the material was calculated as the ratio of minimum and maximum stress after 2 ​min of 20% strain.

#### 2.1.4 Rheological characterization

Photo rheology experiments on the hydrogel precursor solutions (GelMA DOF 50 and DOF80, norHA MMP and norHA DTT) to determine the crosslinking kinetics was measured using a DHR2 rheometer (TA Instruments, The Netherlands). Samples were prepared fresh (100 ​μl measuring volume, n ​= ​3) before loaded onto the rheometer for oscillatory time sweep experiments (frequency of 1.0 ​Hz, angular frequency of 6.28 ​rad/s, with 5.0% constant strain at 37C, preset measuring gap size of 300 um, 20.0 ​mm parallel EHP stainless steel plate). After 30 ​s of measuring, the visible light source was turned on to allow photo crosslinking of the hydrogels for the remaining time of the experiment and storage (G') and loss moduli (G'′) were recorded.

### *2.2* *In vitro* hMSC migration assay

#### 2.2.1 hMSC isolation and expansion

hMSC were isolated from bone marrow of patients undergoing total hip replacement after informed consent (approved by the local Medical Ethical Committees of Erasmus MC: protocol MEC-2015–644) as described earlier [46]. hMSCs were thawed, and expanded in media composed of alpha-Minimum Essential Medium (α-MEM, Gibco, California, USA) supplemented with 10% fetal bovine serum (FBS, Gibco, California, USA), 50 ​μg/mL gentamycin (Gibco), 1.5 ​μg/mL fungizone (Gibco), 1 ​ng/mL fibroblast growth factor 2 (FGF2, AbD Serotec, Puchheim, Germany) and 25 ​μg/mL ascorbic acid-2-phosphate (AA-2-P, Sigma–Aldrich) in a humidified atmosphere with media replacement twice per week. Cells at 80–90% confluency were sub-cultured using 0.25% Trypsin/1x EDTA (Gibco). hMSCs were fluorescently labelled according to the manufacturer's instructions (Vybrant CFDA-SE Cell tracer Kit, Thermo Fisher) to visualize cell location after seeding on the hydrogels (see 2.2.2).

#### 2.2.2 *In vitro* migration assay set up

hMSC migration was evaluated by measuring the migration area of the cells in a 3D migration assay after 48h of culture *in vitro* [47]. Micro-moulds (Micro Tissues 3D Petri Dish, Sigma Aldrich) were casted with agarose to form 256 circular micro-wells. After the micro-moulds gelled, they were transferred to a 12-well-plate containing α-MEM (Gibco) supplemented with 10% foetal bovine serum (FBS, Gibco) and 25 ​μg/mL ascorbic acid-2-phosphate (Sigma-Aldrich) and incubated in a humidified atmosphere (37 ​°C, 5% CO_2_) for 1h. Cell spheroids (500 ​cells per spheroid) were prepared by dropwise seeding the CFDA labelled cell suspension (0.128 ​× ​10^6^ ​cells/190 ​μL) into the 3D agarose moulds and then cultured in a humidified atmosphere for 24 ​h to form spheroids. Spheroid formation was assessed by a standard inverted microscope and irregular sized spheroids were discarded. To harvest the spheroids, the 3D agarose moulds were transferred and inverted into a new 12-well plate containing media and cells and were centrifuged (5 ​min at 120g). The medium containing the spheroids was transferred to a falcon tube and again centrifuged (30 ​s at 300 ​g). Spheroids were resuspended in the assay media composed of α-MEM supplemented with 1% insulin, transferrin and selenium (ITS+, Sigma Aldrich), and 25 ug/mL ascorbic acid-2-phosphate (Sigma-Aldrich) Next, 125 ​μl of each hydrogel precursor was casted in each well of a chamber slide (Nunc cell culture imaging 8-wellls, Thermo Fisher), crosslinked and washed three times with serum free α-MEM. Afterwards, 5–10 spheroids were seeded on each hydrogel in each well. Cell spheroids were cultured on the hydrogels for 48h with assay media supplemented with platelet-derived growth factor BB (50 ​ng/ml, PDGF-BB, Peprotech, NJ, USA) to stimulate cell migration. To quantify cell migration, confocal imaging (Leica SP5, FITC channel, 10x magnification) was performed with the acquisition of z-stacks to monitor the spheroid migration from the top to the bottom of the hydrogel. Cell migration area of hMSC was measured on the different hydrogel compositions (n ​= ​6, n ​= ​5 for THA-col) using the earlier described macro with Fiji image processing software [47]. The migration area of the migrating cells was calculated to obtain the total migratory area in a radius of 760 ​μm.

### 2.3 *Ex vivo* migration assay to assess chondrocyte migration

#### 2.3.1 Cartilage ring isolation and *ex vivo* explant culture

Cartilage explants were isolated from the patellar groove of bovine knee joints (six months old calves, Angst AG, Switzerland) using a biopsy punch (8 ​mm, KAI medical, Arnold Bott AG, Opfikon, Switzerland). Cartilage was separated from the subchondral bone with a scalpel. Explants were washed with DMEM HG (Gibco) supplemented with 1% antibiotics (10 U/ml penicillin, 10 ​μg/ml streptomycin, Gibco). After defect creation (4 ​mm, KAI medical, Arnold Bott AG, Switzerland) in the centre of the explant, the defects were filled with the hydrogel precursors (described in Fig. 1), and then gelled in the defect to ensure optimal integration between the hydrogel and the cartilage tissue. All samples were transferred into a 24 well plate (TPP, Trasadingen, Switzerland) containing DMEM HG enriched with 10% FBS, 50 ​μg/ml ascorbic acid and 1% antibiotics. Samples were cultured *ex vivo* for 21 days (37 ​°C, 5% CO_2_) with media change every 2–3 days.

#### 2.3.2 Cell viability staining of *ex vivo* samples

After 21 days of culture, metabolic activity of cartilage explants was assessed with 3-(4,5-dimethylthiazol-2-yl)-2,5-diphenyltetrazoliumbromid (MTT, 1 ​mg/ml, 1h incubation at 37 ​°C, Sigma Aldrich). Samples were imaged with bright field microscope (Zeiss). Samples were also stained for live-dead assessment (Sigma Aldrich) with Ethidium homodimer (4 ​μM) and Calcein-AM (2 ​μM) according to manufacturer's instructions, washed with PBS after 25 ​min incubation and kept in culture media for microscopic assessment (Zeiss confocal microscope, LSM800).

#### 2.3.3 Histological processing, staining and scoring of *ex vivo* samples

Samples were harvested at day 21, fixed with formalin (4%, Formafix AG, Hittnau, Switzerland), washed with PBS, immersed with sucrose (150 ​mg/ml and 300 ​mg/ml, Sigma-Aldrich), embedded in freezing media (Leica, Nussloch, Germany) and snap frozen with liquid nitrogen. Sections of 8 ​μm were cut with a cryostat microtome (HM 500 OM; Zeiss) and stored at −20 ​°C.

Safranin-O staining was performed to visualize proteoglycans. Cryosections were washed with deionized water to remove freezing media, incubated for 10 ​min with Weigert's hematoxylin (Sigma Aldrich), blued with tap water and incubated with fast green (0.02%, 6 ​min, Sigma Aldrich). After washing with acetic acid (1%, Fluka), slides were incubated in safranin-O (0.01%, 15 ​min, Sigma Aldrich) and differentiated with ethanol (70%, Alcosuisse, Rüti bei Büren, Switzerland) with subsequent dehydration (ethanol 96%, ethanol absolute, Xylene) and cover slipping (Eukitt, Sigma Aldrich). Bright-field microscopic images (Olympus BX63, Olympus) were acquired for cell migration analysis.

**Figure 2:**
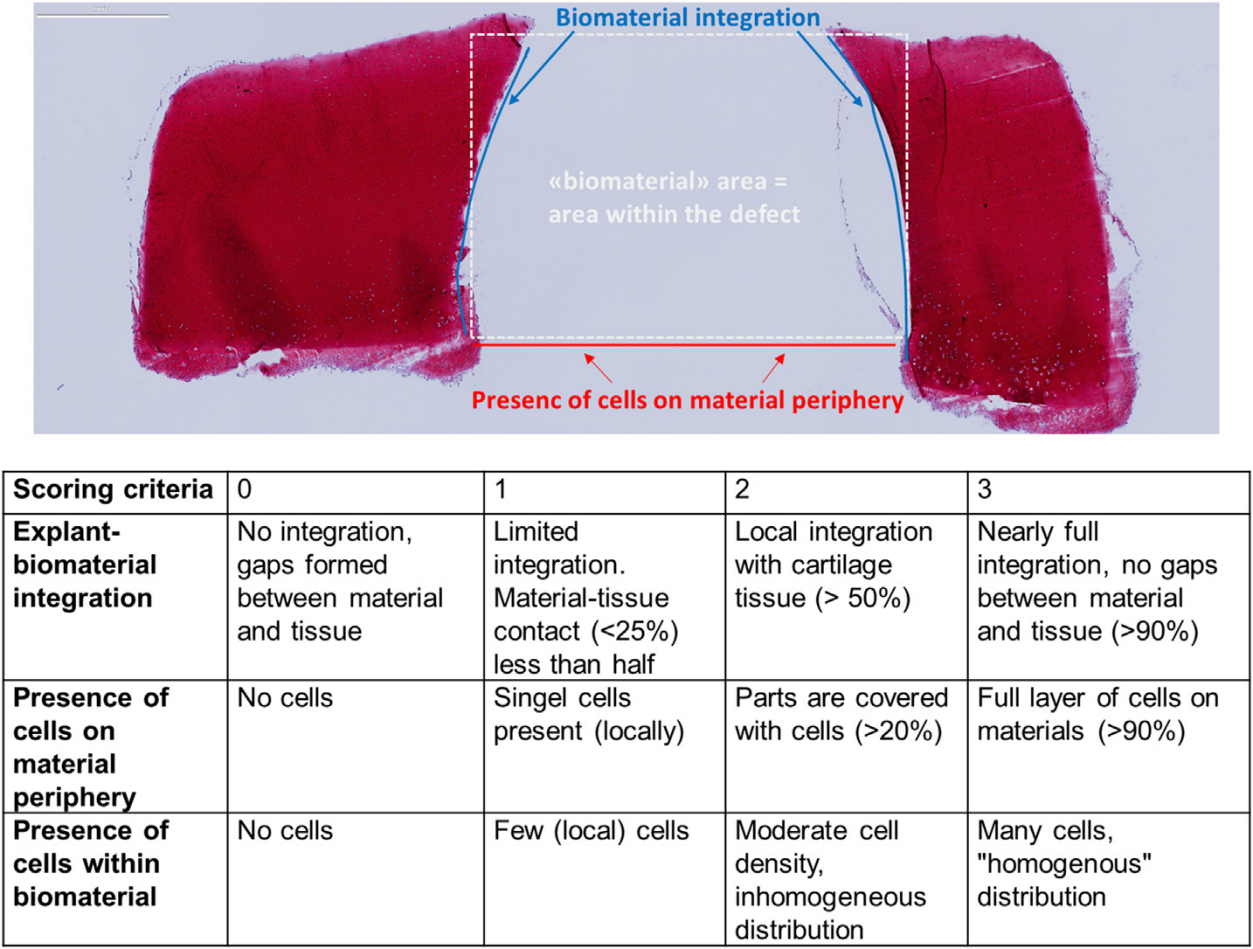
**Scoring criteria to assess chondrocyte migration in the *ex vivo* cartilage ring model.** Safranin-O staining of cartilage explant with defect in the centre (white dashed line). The areas of interest are indicated for the biomaterial integration (blue line) and presence of cells (red line). (For interpretation of the references to color in this figure legend, the reader is referred to the Web version of this article.)

A migration score was introduced to semi-quantitatively evaluate chondrocyte migration from the explant towards the acellular hydrogels. Safranin-O stained slices of the samples at day 21 (n ​= ​3 samples per biomaterial, n ​= ​2 samples empty defect control, n ​= ​2 sections) were scored by three independent observers blinded for the condition (Fig. 2). The individual scores were visually inspected and in case of differences, discussed to reach consensus between observers.

### 2.4 Semi-orthotopic model to assess osteochondral defect repair *in vivo*

#### 2.4.1 *In vivo* subcutaneous osteochondral defect model

To evaluate the capacity of the hydrogels to support endogenous cell migration and osteochondral repair, an *in vivo* semi-orthotopic osteochondral defect model was used, where tissues are implanted subcutaneously in mice [48]. This animal experiment was approved by the ethics committee for laboratory animal use (under license AVD101002016691, protocol #EMC16-691-07) and following the ARRIVE (Animal Research: Reporting of In Vivo Experiments) guidelines. Bovine osteochondral biopsies (8 ​mm diameter, 5 ​mm height) were harvested with a dental trephine from metacarpal-phalangeal joints of 3–8 months old calves from a local slaughterhouse and provided by LifeTec Group (Eindhoven, Netherlands). Osteochondral defects (4 ​mm diameter, 4 ​mm depth) were created in the centre of the biopsies using a hand drill to avoid thermal damage. The defect created into the subchondral bone will allow bone marrow and subchondral bone hosting cells to infiltrate the defect area. The osteochondral biopsies were kept overnight in α-MEM (Gibco, USA) supplemented with 10% fetal bovine serum (FBS, Gibco), 1.5 ​μg/mL fungizone (Gibco), and 50 ​μg/mL gentamycin (Gibco) to ensure sterility. The hydrogel precursors were used to fill the defects and then photo crosslinked before implantation. All hydrogel-loaded osteochondral constructs were covered with a circular 8 ​mm Neuro-Patch membrane (Braun, Melsungen, Germany) on the cartilage to prevent the ingrowth of host cells.

Thirteen 11-week-old female NMRI-Foxn1 nu/nu mice (Janvier Labs, St. Berthevin, France) were used in this study. The mice were allowed to adapt to the conditions of the animal facility for seven days before implantation surgery. The mice were housed under specific-pathogen-free conditions with a regular day/night light cycle and food and water were available ad libitum. Four osteochondral constructs were randomly implanted subcutaneously on the back of each mouse under 2.5–3.0% isoflurane anaesthesia (Laboratories Karizoo, Barcelona, Spain). To minimise the risk of infection, the mice received 25 ​mg/kg bodyweight of ampicillin (Dopharma, Raamsdonksveer, Netherlands) subcutaneously during surgery. Staples (AgnTho's, Lidingö, Sweden) were used to close the incisions and were removed one week after implantation. To ensure pre- and postoperative analgesia, the mice received a subcutaneous injection of 0.05 ​mg/kg body weight of buprenorphine (Chr. Olesen & Co, Copenhagen, Denmark) 1 ​h before surgery and 6 ​h after surgery. After ten days (n ​= ​3 samples per condition) and six weeks (n ​= ​5 samples per condition), mice were killed by cervical dislocation and the osteochondral constructs were harvested.

#### 2.4.2 Histological processing, staining and scoring

The osteochondral constructs were fixed in 4% formalin for 1 week, followed by decalcification using 10% ethylenediaminetetraacetic acid (EDTA, pH 7.4, Sigma Aldrich) for up to 4 weeks. Six-week samples were processed for routine paraffin embedding and sectioned (microtome, Leica). Dewaxed slides were stained with hematoxylin and eosin (HE) to study general cell and tissue morphology. Images were taken with a slide scanner (NanoZoomer, Hamamatsu). Ten-week samples were processed for cryo embedding (OCT, Sakura, Nagano, Japan) and cutting (Cryostat, Leica, Nussloch, Germany) after demineralization.

Image analysis (NDP.View2 software, version 2.8.24, 2020 Hamamatsu Photonics K.K.) was used to assess tissue formation. The area of the newly formed tissue was measured by manually selecting the tissue regions. The percentages of the defects covered with cartilage-like, bone-like and other tissue were measured separately (supplementary Figure S4D), with the defect area set to 100%. The tissue volume of three sections that were taken at depths of 1, 1.5 and 2 ​mm for each sample was averaged for further analyses. All slides were scored by an investigator blinded to the experimental condition.

### 2.5 Statistical analysis

Mechanical characterization and *ex vivo* migration are presented as box plots (min to max value with a line presenting the median). *In vitro* MSC migration area and tissue volume in % are presented as mean ​± ​standard deviation (SD). For each of the hydrogel groups (THA, GelMA, norHA) the results of the two modifications were compared. All statistical analyses were performed using SPSS (version 28.0.1.0, IBM Corporation, USA). Student t-tests were performed for mechanical characterization, *in vitro* MSC migration and *in vivo* tissue formation and two-sided p-values are reported. In case the variances between the two modifications were significantly different, p-value of equal variances not assumed is reported and marked with a ∗. Mann–Whitney U tests were used to assess statistical significance in the *ex vivo* migration scores. For all statistical analyses, a p-value <0.05 was considered statistically significant and marked with a ∗.

## 3 Results

### 3.1 Lower crosslinking density of GelMA hydrogels increases cell migration

**Figure 3:**
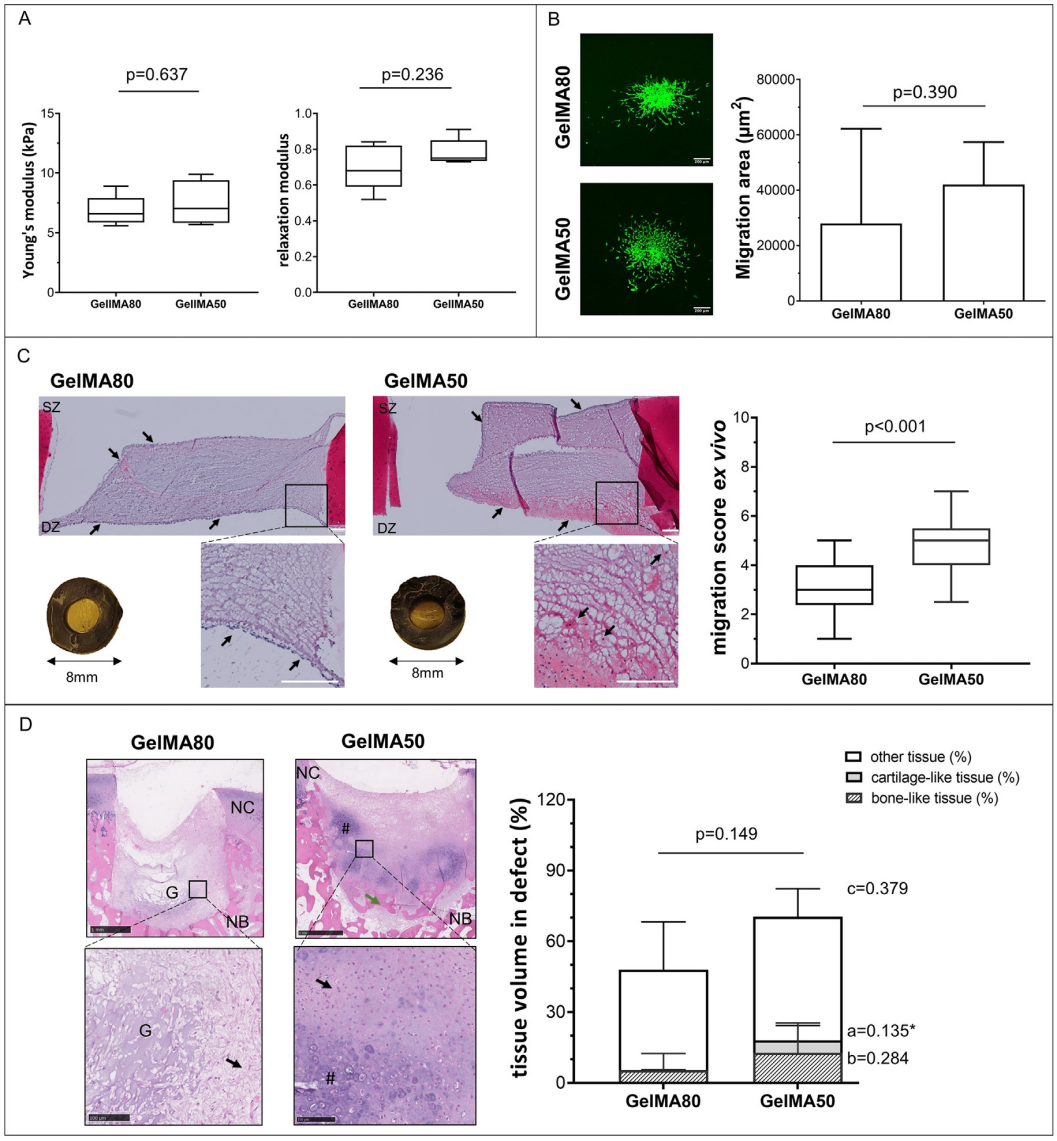
**Effect of lowering the degree of functionalization (DoF) from 80% to 50% in gelatine methacryloyl (GelMA) hydrogels on cell migration and tissue formation.** A) the Young's modulus and stress-relaxation to characterize hydrogel recovery after swelling in PBS (n ​= ​5 per group). B) *In vitro* cell migration of hMSCs out of spheroids seeded on top of hydrogels (n ​= ​6 per group). hMSC labelled with membrane dye are shown in green. Scale bar 200 ​μm. C) Chondrocyte migration and migration score in the *ex vivo* cartilage ring model (min to max score with median). Macroscopic (explant diameter 8 ​mm) and microscopic image of MTT stained samples, the safranin-O stained section of the hydrogel and migration score with a maximum score of 9 (n ​= ​3 samples per group, n ​= ​2 slides per sample). Scale bar 200 ​μm. D) Cell migration and related tissue formation in acellular hydrogels implanted in osteochondral explants in a semi-orthotopic mouse model (n ​= ​5 samples per biomaterial). Hematoxylin and eosin-stained cross-sections of one representative sample are shown. Tissue volume in osteochondral defect (OCD) is presented as bone-like tissue, cartilage-like tissue and other tissue (mean ​± ​SD) relative to total tissue volume. Scale bar low magnification image 1 ​mm, higher magnification image 100 ​μm. NC: native cartilage, NB: native bone, G: hydrogel, # indicated newly formed cartilage-like tissue, green arrow indicates newly formed bone-like tissue, black arrow indicates infiltrated cells within the defects, blue arrow indicates the integration of newly formed bone and native bone. Statistical significance comparing means between hydrogel formulations: a: cartilage-like, b: bone-like, c: other tissue, p: total tissue volume. ∗ equal variances not assumed. (For interpretation of the references to color in this figure legend, the reader is referred to the Web version of this article.)

Compression moduli (E-moduli) of GelMA hydrogels with DoF of 50% and 80% crosslinked under the same conditions after equilibrating in PBS were respectively 6.8 ​± ​1.3 ​kPa and 7.3 ​± ​1.7 ​kPa, p ​= ​0.637, revealing no significant difference between the two DoF (Fig. 3A). Stress recovery of GelMA80 (0.8 ​± ​0.1) was similar to that of GelMA50 (0.7 ​± ​0.1, p ​= ​0.236).

The ^1^H NMR spectrum confirms the different DOF (Supplementary Figure S1). GelMA50 had slower photo-crosslinking kinetics than GelMA80, both with a final storage modulus around 1 ​kPa (Supplementary Figure S2).

hMSCs migrated out of the spheroid in both GelMA formulations. The migration area (Fig. 3B) of hMSC seeded in GelMA50 (42,050 ​± ​15,335 ​μm^2^) was similar to the migration in GelMA80 (28,010 ​± ​34,241 ​μm^2^, p ​= ​0.390∗) trending towards more migration in the lower DoF. In the *ex vivo* migration assay, the chondrocytes in the cartilage explant remained metabolic active (as indicated by MTT staining Fig. 3C) and a few metabolically active chondrocytes invaded both GelMA formulations (supplementary Figure S6A). Most of the cells were observed on top of the hydrogels. GelMA50 had a significantly higher chondrocyte migration score (min/max:2.5/7, median:5) than GelMA80 (min/max:1/5, median:3, p ​< ​0.001). The hydrogels with both DoF remained inside the cartilage defect. In general, a slight shrinkage was observed during culture resulting in a gap between the hydrogel and the surrounding defect.

Hydrogels were implanted in the semi-orthotopic *in vivo* model. After ten days, infiltration of a few multinucleated cells was observed in GelMA80 (2 out of 3 samples) and GelMA50 (1 out of 3 samples), indicating a low inflammatory response (supplementary Figure S3). After six weeks the average area of remaining hydrogel implanted in the osteochondral defect was 11.3% ​± ​9.7% for GelMA50 and 16.4% ​± ​12.3% for GelMA80 (values calculated relative to respective total defect area). This means that the hydrogel was degraded upon implantation. The infiltration of cells and newly formed osteochondral tissue were mostly present in the deep zone and at the lateral sides of the osteochondral defects (Fig. 3D). In GelMA80 a cell-free area within the hydrogel was still present, even in the samples with the highest invasion (see supplementary Figure S4A and S5A). GelMA50 and GelMA80 both allowed osteochondral defect repair (70.5% ​± ​21.3% vs 48.0% ​± ​23.0%, p ​= ​0.149). The repair consisted of cartilage-like tissue (0.04% ​± ​0.09% vs 5.3% ​± ​6.3%, p ​= ​0.135∗), bone-like tissue (5.4% ​± ​7.1% vs 12.8% ​± ​12.6%, p ​= ​0.284) and other tissues (52.4% ​± ​11.9% vs 42.6% ​± ​20.2%, p ​= ​0.379).

### 3.2 MMP cleavable norHA hydrogels limit cell migration *in vitro* and *ex vivo* but increase tissue formation *in vivo*

**Figure 4:**
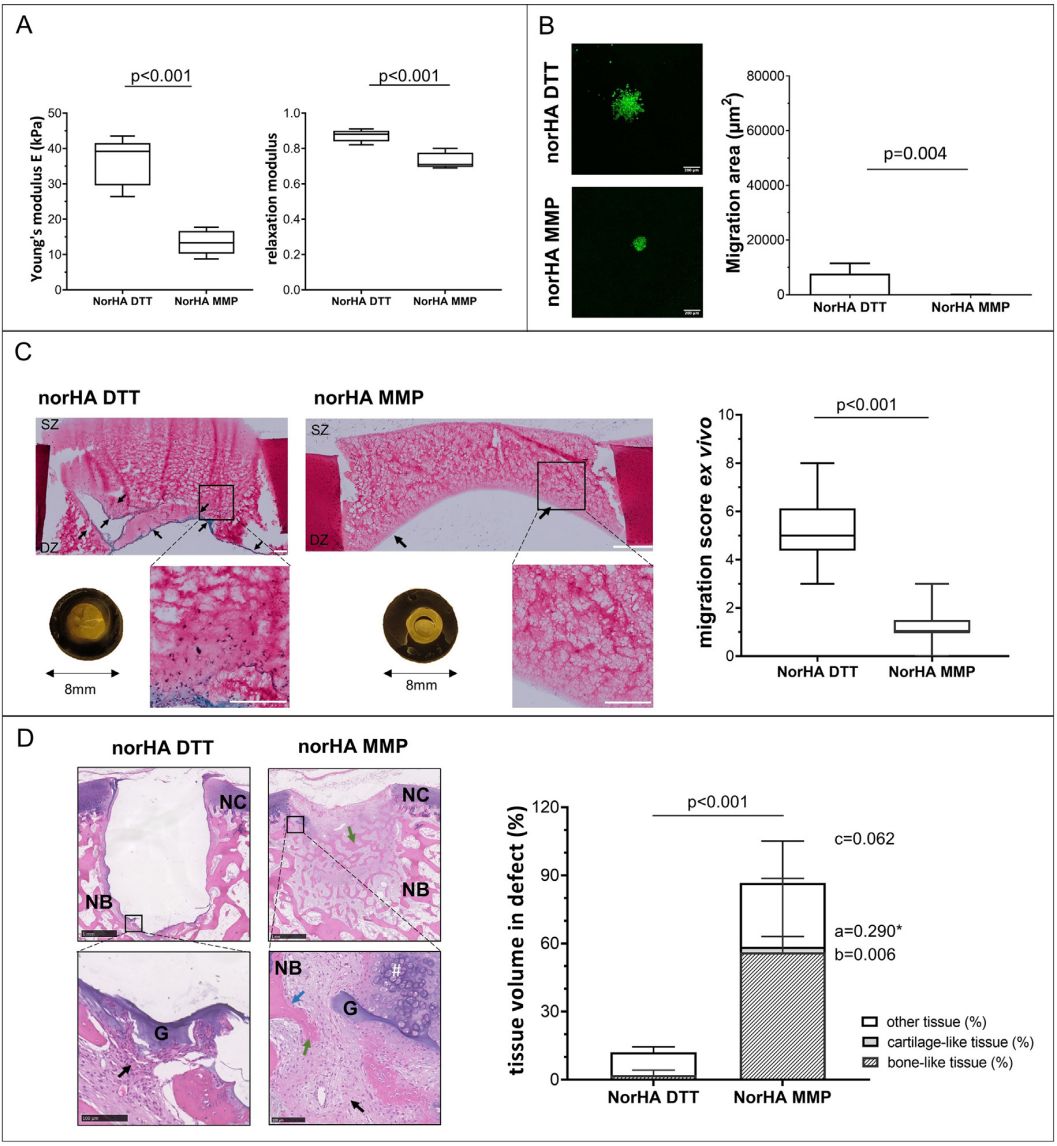
**Effect of crosslinker degradability (MMP degradable vs non-degradable DTT) on norbonene modified hyaluronic acid hydrogel (norHA) cell migration and tissue formation.** A) Young's modulus and stress-relaxation to characterize hydrogel recovery after swelling in PBS (n ​= ​5 per group) are shown. B) *In vitro* cell migration of hMSCs out of spheroids seeded on top of hydrogels (n ​= ​6 per group). hMSC labelled with membrane dye are shown in green. Scale bar 200 ​μm. C) Chondrocyte migration and migration score in the *ex vivo* cartilage ring model (min to max score with median). Macroscopic (explant diameter 8 ​mm) and microscopic image of MTT stained samples, the safranin-O stained section of the hydrogel and migration score with a maximum score of 9 (n ​= ​3 samples per group, n ​= ​2 slides per sample). Scale bar 200 μm low magnification 500 ​μm. D) Cell migration and related tissue formation in acellular hydrogels implanted in osteochondral explants in a semi-orthotopic mouse model (n ​= ​5 samples per biomaterial). Hematoxylin and eosin-stained cross-sections of one representative sample are shown. Tissue volume in osteochondral defect (OCD) is presented as bone-like tissue, cartilage-like tissue and other tissue (mean ​± ​SD) relative to total tissue volume. Scale bar low magnification image 1 ​mm, higher magnification image 100 ​μm. NC native cartilage, NB native bone, G: hydrogel, green arrow indicates newly formed bone-like tissue, black arrow indicates infiltrated cells within the defects, blue arrow indicates the integration of newly formed bone and native bone. Statistical significance comparing means between hydrogel formulations: a: cartilage-like, b: bone-like, c: other tissue, p: total tissue volume. ∗ equal variances not assumed. (For interpretation of the references to color in this figure legend, the reader is referred to the Web version of this article.)

The two norHA hydrogel formulations differed by the presence of either a MMP degradable crosslinker or a non-degradable (DTT) crosslinker. The modulus of norHA MMP (13.4 ​± ​3.5 ​kPa) was significantly lower than the modulus of norHA DTT (36.3 ​± ​6.8 ​kPa, p ​< ​0.001) after swelling and equilibrating in PBS (Fig. 4). The stress recovery was significantly higher in norHA DTT (0.9 ​± ​0.0) compared to norHA MMP (0.7 ​± ​0.1, p ​< ​0.001). The ^1^H NMR spectrum of norHA confirms the functionalization of HA with norbornene groups (Supplementary Figure S1). The results of the photo rheological characterization (Supplementary Figure S2) showed a similar trend to the DMA measurement with a higher storage modulus for norHA DTT (1.5 ​kPa) compared to norHA MMP (0.2 ​kPa).

Both NorHA hydrogel formulations showed limited migration of MSC into the hydrogel *in vitro*. Less hMSC migration (Fig. 4B) was observed when a norHA MMP degradable crosslinker (44 ​± ​68 ​μm^2^) was used, compared to a norHA non-degradable DTT crosslinker (7742 ​± ​3772 ​μm^2^, p ​= ​0.004). NorHA MMP was also invaded by less chondrocytes than norHA DTT after three weeks of *ex vivo* culture (Fig. 4C, supplementary Figure S8A, B). A significanty lower migration score was observed for the norHA material with a degradable crosslinker (norHA MMP, min/max: 0/3, median: 1) compared to norHA with a non-degradable crosslinker (norHA DTT, min/max:3/8, median:5, p ​< ​0.001). Both hydrogel formulations remained in the defect during 21 days of culture.

After ten days of implantation *in vivo* (Fig. 4D) no cell invasion was present in norHA DTT hydrogels (0 out of 3 samples) while the group with the MMP degradable crosslinker exhibited invading cells even in the deeper regions of the hydrogels (3 out of 3 samples (supplementary Figure S3). After six weeks of implantation total tissue formation was significantly higher in norHA MMP than norHA DTT (86.6% ​± ​15.9% vs. 12.1% ​± ​3.7%, p ​< ​0.001). The average percentage of remaining hydrogel in the defect was 0.7% ​± ​0.5% for norHA MMP and 8.5% ​± ​9.7% for norHA DTT (values calculated relative to respective defect area). The infiltrated cells and newly formed tissue were mostly in the deeper parts and at the sides of the osteochondral defects in the periphery of the hydrogels (Fig. 4D). Cartilage-like tissue (2.4% ​± ​4.5% vs 0.0% ​± ​0.0%, p ​= ​0.290∗) was observed with areas of proteoglycan-rich matrix (supplementary Figure S5B). Significantly more bone-like tissue (56.1% ​± ​32.6% vs 2.0% ​± ​2.3%, p ​= ​0.006) and a tendency towards more other-like tissue (28.1% ​± ​18.4% vs 10.1% ​± ​2.4%, p ​= ​0.062) was formed in the norHA MMP compared to norHA DTT.

### 3.3 The addition of collagen fibres to THA improves cell migration *in vitro* and *ex vivo*

**Figure 5:**
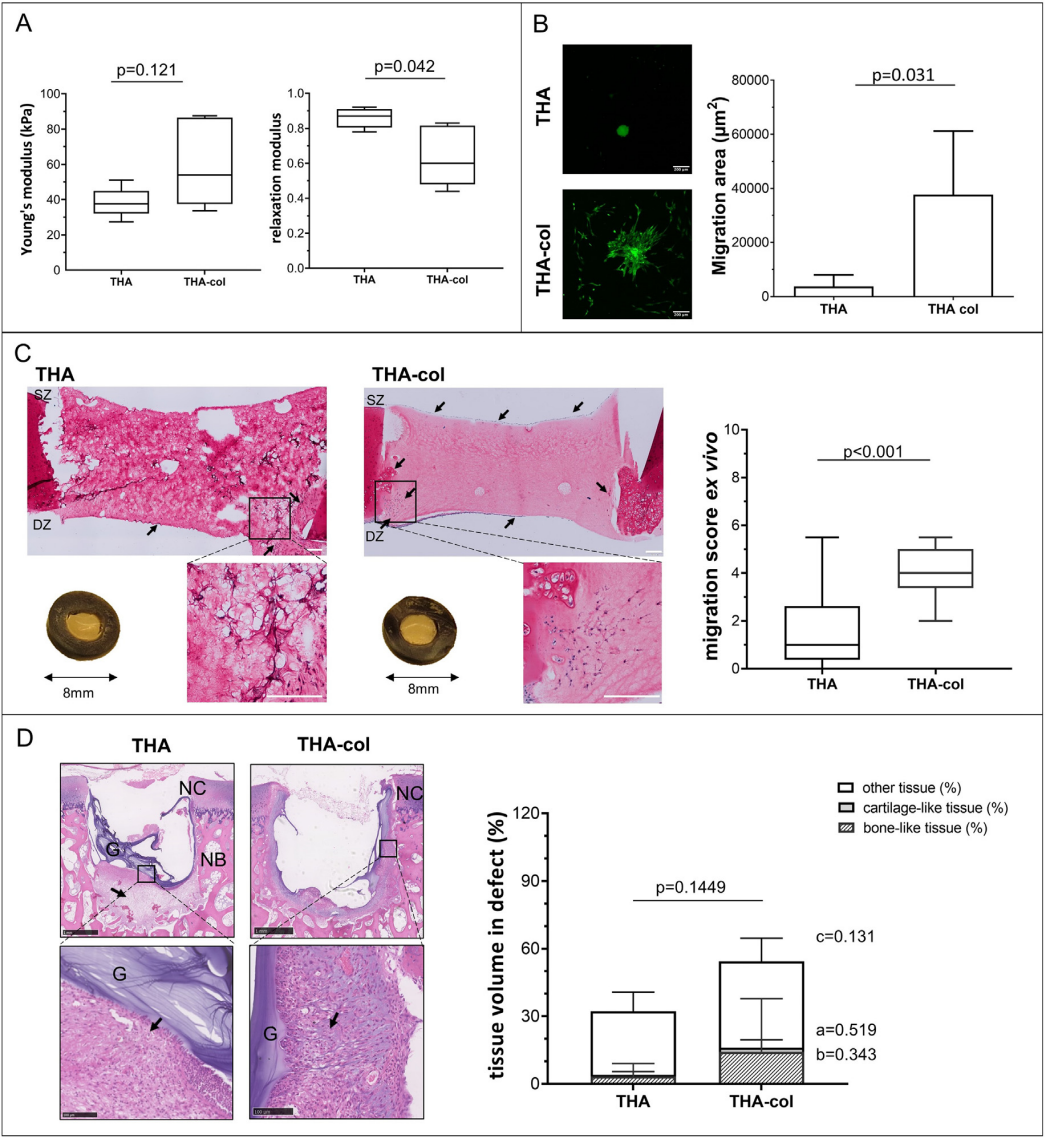
**Effect of the addition of fibrillary collagen (col) to tyramine modified hyaluronic acid (THA) hydrogels on cell migration and tissue formation.** A) Young's modulus and stress-relaxation to characterize hydrogel recovery after swelling in PBS (n ​= ​5 per group). B) *In vitro* cell migration of hMSCs out of spheroids seeded on top of hydrogels (n ​= ​6 THA, n ​= ​5 THA-col). hMSC labelled with membrane dye shown in green. Scale bar 200 ​μm. C) Chondrocyte migration and migration score in the *ex vivo* cartilage ring model (min to max score with median). Macroscopic (explant diameter 8 ​mm) and microscopic image of MTT stained samples, the safranin-O stained section of the hydrogel and migration score with a maximum score of 9 (n ​= ​3 samples per group, n ​= ​2 slides per sample). Scale bar 200 ​μm. D) Cell migration and related tissue formation in acellular hydrogels implanted in osteochondral explants in a semi-orthotopic mouse model (n ​= ​5 samples per biomaterial). Haematoxylin and eosin-stained cross-sections of one representative sample are shown. Tissue volume in osteochondral defect (OCD) is presented as bone-like tissue, cartilage-like tissue and other tissue (mean ​± ​SD) relative to total tissue volume. Scale bar low magnification image 1 ​mm, higher magnification image 100 ​μm. NC native cartilage, NB native bone, G: hydrogel, black arrow indicates infiltrated cells within the defects, blue arrow indicates the integration of newly formed bone and native bone. Statistical significance comparing means between hydrogel formulations: a: cartilage-like, b: bone-like, c: other tissue, p: total tissue volume.∗ equal variances not assumed. (For interpretation of the references to color in this figure legend, the reader is referred to the Web version of this article.)

Moduli (Fig. 5A) of THA-col (60.4 ​± ​24.9 ​kPa) were similar to THA (38.3 ​± ​8.5 ​kPa, p ​= ​0.121). The stress recovery was significantly increased in THA compared to THA-col (0.9 ​± ​0.1 vs 0.6 ​± ​0.2, p ​= ​0.042∗). The ^1^H NMR spectrum of THA confirm the functionalization of HA with tyramine (Supplementary Figure S1).

THA-col had significantly more MSC migration *in vitro* (Fig. 5B) (37,647 ​± ​23,532 ​μm^2^) than THA only (3737 ​± ​4243 ​μm^2^, p ​= ​0.031∗). Only a few metabolic active chondrocytes were present in the THA hydrogel, and more cells were present in THA-col hydrogel in the *ex vivo* model (Fig. 5C, supplementary Figure S6A). This was also visible from the chondrocyte migration score that was significantly higher in THA-col (chondrocyte migration score min/max; 2/5.5, median 4) than in THA (min/max: 0/5.5, median 1, p ​< ​0.001). Hydrogels of both formulations remained in the cartilage defect during the 21 days of culture.

Ten days after implantation *in vivo*, a few inflammatory cells were observed on the hydrogels (THA: 2 out of 3 samples, THA-col: 2 out of 3 samples, supplementary Figure S3), located at the hydrogel periphery and inside the hydrogels. After six weeks of implantation, the average amount of remaining hydrogel in the defect was 9.7% ​± ​3.0% for THA-col and 15.2% ​± ​9.1% for THA (values calculated relative to respective defect area). For THA materials, limited cell migration and total osteochondral tissue repair (THA-col: 54.4% ​± ​26.4% vs. THA: 32.3% ​± ​15.4%, p ​= ​0.144) was observed in the defects of the osteochondral explants. In line with the *in vitro* hMSC migration and *ex vivo* chondrocyte migration, a trend for an increased amount of cartilage-like tissue (1.8% ​± ​3.5 vs 0.7% ​± ​1.5%, p ​= ​0.519), bone-like tissue (14.3% ​± ​23.6% vs 3.4% ​± ​5.7%, p ​= ​0.343) and other tissue (38.3% ​± ​10.3% vs 28.3% ​± ​8.4% p ​= ​0.131) were observed in the defects filled with THA-col composite compared to THA alone (Fig. 5D, supplementary Figure S4C and S5C).

## 4 Discussion

Material-based cell-free approaches to treat cartilage and osteochondral defects have shown promising results [5,49,50]. These approaches are of interest due to the limited availability of autologous chondrocytes. Infiltration of cells from the adjacent tissues into the biomaterial is a critical step in this approach, as these migrating cells play a pivotal role in depositing the extracellular matrix and facilitating tissue repair [15]. However, data on which biomaterial properties influence cell infiltration and related tissue formation is limited since most studies use only one material or modification, and different studies use different models for their evaluation. Focus of this study was to evaluate different materials and modifications in an *in vitro* assay for MSC migration, an *ex vivo* assay for chondrocyte migration and an *in vivo* model to assess cell infiltration, inflammatory response and tissue formation. Our findings indicate significant differences between the three hydrogel groups across the various assays, and the outcomes may be extended to other materials and modifications.

Lowering DoF from 80% to 50% (GelMA80 vs. GelMA50) showed a higher migration index and thus a higher number of chondrocytes migrating into the hydrogel *ex vivo*. It has also been described that the hydraulic permeability and mechanical properties of GelMA can be controlled by the DoF, polymer concentration and crosslinking time [51]. The mechanical behaviour of the two formulations during the rheology tests was not significantly different and did not allow estimation of the permeability of the gels. The lower degree of functionalization of GelMA50 has been previously associated with a faster enzymatic degradation kinetic [20], which is known to promote cell migration. A higher cell migration and more tissue formation compared to the higher DoF group was only observed in the *ex vivo* migration assay. The MSCs in the *in vitro* assay were spreading and migrating in both, GelMA50 and GelMA80. This partially aligns with a previous *in vitro* study comparing GelMA (5% wt) with different DoF, which showed spreading of encapsulated adipose-derived MSCs in the samples with a DoF of 30% and 50%, but not above 70% [21,43]. In the *ex vivo* assay, the difference in migration score between the two DoF was more prominent. Although the chondrocytes retained a round morphology, they seem to prefer invasion into the lower DoF hydrogel. This finding highlights that different models and/or the cell types used, influence migration behaviour. A tendency of overall more tissue formation was observed in GelMA50 compared to GelMA80 after six weeks of implantation. Although GelMA has some potential to stimulate cell migration and cartilage-like-tissue formation, the shrinking of the hydrogel when cultured in the cartilage ring model is a drawback. Nguyen et al. described that GelMA with a lower DoF degrades faster by collagenase-loaded micro-particles [19]. This shrinkage leads to limited integration with the surrounding tissue and resulting in gap formation that cells might not be able to bridge.

A different approach to control the degradability of a hydrogel and to increase cell migration is the use of a MMP degradable crosslinker with a norHA hydrogel. In contrast to our original hypothesis, that the use of a MMP cleavable crosslinker would improve cell migration, the addition of the MMP degradable crosslinker did not improve cell migration *in vitro* or in the *ex vivo* setting. It is possible that the proteases secreted by the MSCs and the cartilage explants in these settings were not capable of sufficiently degrading the VPMSMRGG peptide of the crosslinker in our study. It has been investigated before that the VPMSMRGG is most sensitive to MMP1 [52,53]. It is likely that there are more MMPs present in the *in vivo* model that lead to a faster degradation. In addition, it is plausible that the *ex vivo* model prompts the secretion of tissue inhibitors of matrix metalloproteinases, which hinder the activity of MMPs and, as a result, impede the degradation of the hydrogel [54,55]. Although no differences in MSCs [41], and (lung) epithelial cells [56] were observed in previous studies, future studies will need to investigate whether specific MMPs are responsible to degrade these bonds in the norHA-MMP gels. Furthermore, the more than three-fold lower bulk mechanical properties of norHA-MMP could have limited cell attachment and thus migration. In the *in vivo* model, however, the MMP-sensitive crosslinker clearly showed more tissue formation in the gels crosslinked with the non-degradable DTT crosslinker. The formation of various tissue types, including cartilage-like and bone-like tissue, in the semi-orthotopic model aligns with prior literature, which has reported that in norHA hydrogels that incorporate non-degradable crosslinkers, matrix deposition is confined to the pericellular region after 28 days [27]. Additionally, research on malemeide modified HA (1.2% wt) crosslinked with a MMP-sensitive crosslinker (CRDVPMSQMRGGDRCG) has shown that a greater amount of cartilage-like tissue is formed compared to gels with protease-insensitive DTT crosslinkers [57]. Thus, our study verifies the benefit of using MMP-cleavable crosslinker to promote the formation of repair tissue and cartilage-like matrix deposition. Furthermore, our study underscores the importance of utilizing multiple assays and selecting an appropriate experimental design to evaluate the performance of a hydrogel.

Besides using a MMP degradable crosslinker, the addition of RGD peptides is another approach to control cell spreading. Collagen type I and, to some extent, GelMA contains natural RGD sites that are characterized by cell–instructive properties [58]. We found that the addition of collagen in a THA hydrogel enhanced the migration of MSCs and chondrocytes in all three models examined. Furthermore, the incorporation of type 1 collagen into the THA hydrogel leads to the development of a fibrillary network, which induces bio-instructive properties sensed by the cells, thereby providing an additional advantage. It has been shown that these micro-structural features in THA-col can be used to orient collagen fibres via 3D bioprinting [39]. Whether the fibrillar component, the RGD sites or a combination of both are the most driving factor promoting cell migration remains unknown and a focus for future studies. Although THA-col composite did positively influence MSC migration and MSC chondrogenesis *in vitro* [59,60], still only 60% of the defect volume was filled with newly formed tissue, and limited cartilage-like tissue was formed in the *in vivo* setting. Further optimization of this hydrogel is needed to achieve more cell invasion and support cartilage-like tissue formation.

Cell-material interaction and thus mechanosensing contributes to cell spreading and migration. The results suggest that cell migration is not primarily controlled by the bulk mechanical properties as it has been proposed in other studies. It is rather a combination of different material properties that control cell behaviour [47,61,62]. THA and THA-col had similar Young's moduli and the higher cell migration in the THA-col compared to THA is likely due to the presence of RGD sequences. Storage moduli of THA and THA-col have been shown to be similar [34,39]. GelMA80 and GelMA50 both naturally contain RGD sequences and in this study the young's moduli as well as final storage moduli were comparable [43]. Yet, migration on GelMA50 was better in the *ex vivo* model compared to GelMA80. This indicates that the lower DOF can have a positive effect on cell migration and tissue formation. Both norHA hydrogels contained RGD for cell adhesion. In the *in vitro* and the *ex vivo* model, more cell invasion was found in the stiffer norHA DTT hydrogel compared to norHA MMP. Caliari et al. have shown that MSC spreading depends on the substrate stiffness of norHA hydrogels, and found that stiffer hydrogels (20 ​kPa) stimulated MSC spreading in 2D, whereas in 3D cell spreading was seen only in the least stiff hydrogel (1 ​kPa) [36]. This discrepancy with our results in norHA gels indicates that the dimensionality of the model and hydrogel stiffness are not the only parameters influencing cell migration. Instead, a complex interplay of multiple parameters might affect cell migration in hydrogels. More research is needed to understand the underlying mechanisms of bioinstructive properties to increase cell infiltration and improve biochemical and biophysical properties of biomaterials for cell free tissue repair.

Evaluating the samples implanted for only ten days in the semi-orthotopic mouse model, we identified only a few inflammatory cells on and within the hydrogels, highlighting that there was no strong foreign body reaction related to any of the hydrogels used. Although a strong foreign body response can cause unwanted effects, this inflammation is also known to influence cell migration as well as matrix degradation and therefore can be stimulating the ingrowth of cells in a hydrogel [7,14,63,64]. In the design and modification of biomaterials such a positive effect of inflammation should be considered as well. To pre-screen biomaterials and modifications on their pro- or anti-inflammatory response, different *in vitro* models are available. Wesdorp et al. showed a similar response of neutrophils seeded on THA, THA-col (2.5% wt THA, 0.25% wt col) and GelMA (15% wt, DoF 50%) in terms of myeloperoxidase, neutrophil elastase and cytokine secretion [65]. In light of the 3R principle (refinement, reduction, and replacement), an initial screening may be conducted *in vitro* to assess the potential impact of hydrogel modifications on inflammation, which could alter the cell infiltration behavior of the hydrogel.

Cell type specific differences in response to the materials as a function of the mechanical properties, chemical composition and mesh size have been reported [26,66,67]. The screening of the hydrogels in the three models covers different cell types, namely MSCs, chondrocytes and cells residing in the subchondral bone and bone marrow of the osteochondral explants. Results of the here reported *in vitro, ex vivo* and *in vivo* testing together with findings related to how cells respond to different materials suggest that the specific hydrogel formulations tested in this study were not selective for different mesenchymal cell types present in the osteochondral environment. This study further showed that with a single modification in either a HA or gelatine-based hydrogel, cell invasion can be stimulated. A limitation of the present study is that for each formulation multiple characteristics of the matrices (total polymer concentration, crosslinking density, mesh size) are simultaneously modified, limiting the ability to decouple findings. Since each modification will have different effects on hydrogel swelling, secondary structure, degradation and most likely the cell–biomaterial interaction, a direct comparison would have been not very practical. The current approach prevented this direct comparison and solely demonstrated the possible effects of one specific chemical modification within three selected hydrogel types on cell migration and tissue formation. More fundamental knowledge on how different material properties influence cell behaviour is required to optimize the design of hydrogels for osteochondral tissue repair.

The success of acellular hydrogel approaches to repair (osteo-) chondral defects is limited in treating larger defects. In this case, the relatively low number of cells invading the biomaterial implanted in the defect might not allow for complete defect repair. One approach to increase the invasion distance might be the use of chemotactic factors. If this is not feasible, a combined approach of encapsulating patient-derived cells and a hydrogel stimulating the migration and invasion of cells from surrounding tissue can be considered. Moreover, good bonding and minimal lateral delamination of the hydrogel from the surrounding tissue are a prerequisite allowing for endogenous cell migration and related matrix deposition [68]. To address this research question, different models including push out tests are available for quantitative measures of integration [69,70]. DMA has been proposed to study the adhesion strength between the tissue and hydrogel [71]. Before taking this next step, a more in-depth understanding is needed to improve cell invasion further and to understand the mechanisms driving or limiting cell migration.

## 5 Conclusion

Migration of cells with chondrogenic potential into hydrogels is the first step to improve cell free repair of (osteo-) chondral defects. While cell free approaches would fail in the absence of cell invasion, cell-based treatments would also benefit from enhanced integration of the implant to the surrounding tissue. Our study shows that cell migration is dependent on multiple material characteristics, including physicochemical and bio-instructive properties. Moreover, this study also highlights the need for screening biomaterials in different models *in vitro, ex vivo* and *in vivo* since results might differ depending on the model used. The three hydrogel groups and modifications screened in this study, do support cartilage-like and bone-like tissue formation making them suitable candidates for further optimization and use in osteochondral repair.

## Author contribution

**A. Schwab**: Conceptualization and methodology (*ex vivo* model), data analysis (*ex vivo* and *in vivo* study, DMA), interpretation and visualization, writing original draft, manuscript reviewing and editing. **M.A. Wesdorp**: Conceptualization and methodology (*in vitro* and *in vivo* model), data analysis (*in vitro* and *in vivo* study), interpretation, manuscript reviewing and editing. **J. Xu**: Methodology (*in vivo* study), data analysis (*in vivo* study), interpretation and visualization, manuscript reviewing and editing. **F. Abinzano**: Methodology and data analysis (DMA). **F Falandt:** Methodology (photorheology and ^1^H NMR), data analysis, **C Loebel**: providing norHA, manuscript reviewing. **J. Malda, J.A. Burdick**: Supervision, manuscript reviewing. **R Levato**: providing GelMA, supervision, manuscript reviewing. **D. Eglin, M.J. Stoddart**: Conceptualization, supervision, Project administration, Funding acquisition, manuscript reviewing. **R. Narcisi**: Supervision, data interpretation, manuscript reviewing. **M. D'Este**: Providing THA, conceptualization, supervision, project administration, manuscript reviewing. **G.J.V.*M. van* Osch**: Conceptualization, supervision, data interpretation, funding acquisition, manuscript reviewing and editing. All authors approved the final version of the manuscript.

## Ethical approval

All animal studies were performed following the ARRIVE (Animal Research: Reporting of In Vivo Experiments) guidelines for the work with animals and approved by the ethics committee for laboratory animal use under license AVD101002016691, of the Centrale Commissie Dierproeven (CCD) and protocol #EMC16-691-07 of the Erasmus MC Animal Ethical Committee. The isolation of hMSCs from patients undergoing total hip replacement was performed after informed consent and approved by the local Medical Ethical Committees of Erasmus MC (protocol MEC-2015–644).

## Declaration of competing interest

A conflict of interest occurs when an individual's objectivity is potentially compromised by a desire for financial gain, prominence, professional advancement or a successful outcome. The Editors of the *Journal of Orthopaedic Translation* strive to ensure that what is published in the Journal is as balanced, objective and evidence-based as possible. Since it can be difficult to distinguish between an actual conflict of interest and a perceived conflict of interest, the Journal requires authors to disclose all and any potential conflicts of interest.

Section I: The authors whose names are listed immediately below certify that they have NO affiliations with or involvement in any organization or entity with any financial interest (such as honoraria; educational grants; participation in speakers’ bureaus; membership, employment, consultancies, stock ownership, or other equity interest; and expert testimony or patent-licensing arrangements), or non-financial interest (such as personal or professional relationships, affiliations, knowledge or beliefs) in the subject matter or materials discussed in this manuscript.

Author names: A. Schwab, M.A. Wesdorp, J. Xu, F. Abinzano, C. Loebel, M. Falandt.

Section II: The authors whose names are listed immediately below report the following details of affiliation or involvement in an organization or entity with a financial or non-financial interest in the subject matter or materials discussed in this manuscript. Please specify the nature of the conflict on a separate sheet of paper if the space below is inadequate.

Author names and details of the conflict(s) of interest: G.J.V.M. van Osch, J. Malda, R. Levato, J. Burdick, D. Eglin, M.J. Stoddart, R. Narcisi, M. D’Este: The work is performed in the course of a grant of the AO Foundation with money to the account of the employer.
