# Supplementary material for: Modulating design parameters to drive cell invasion into hydrogels for osteochondral tissue formation
Source: J Orthop Translat. 2023 Sep 4;41:42–53. doi: 10.1016/j.jot.2023.07.001 (PMC10485598; doi:10.1016/j.jot.2023.07.001)
Supplement: Multimedia component 1 [file mmc1.pdf]

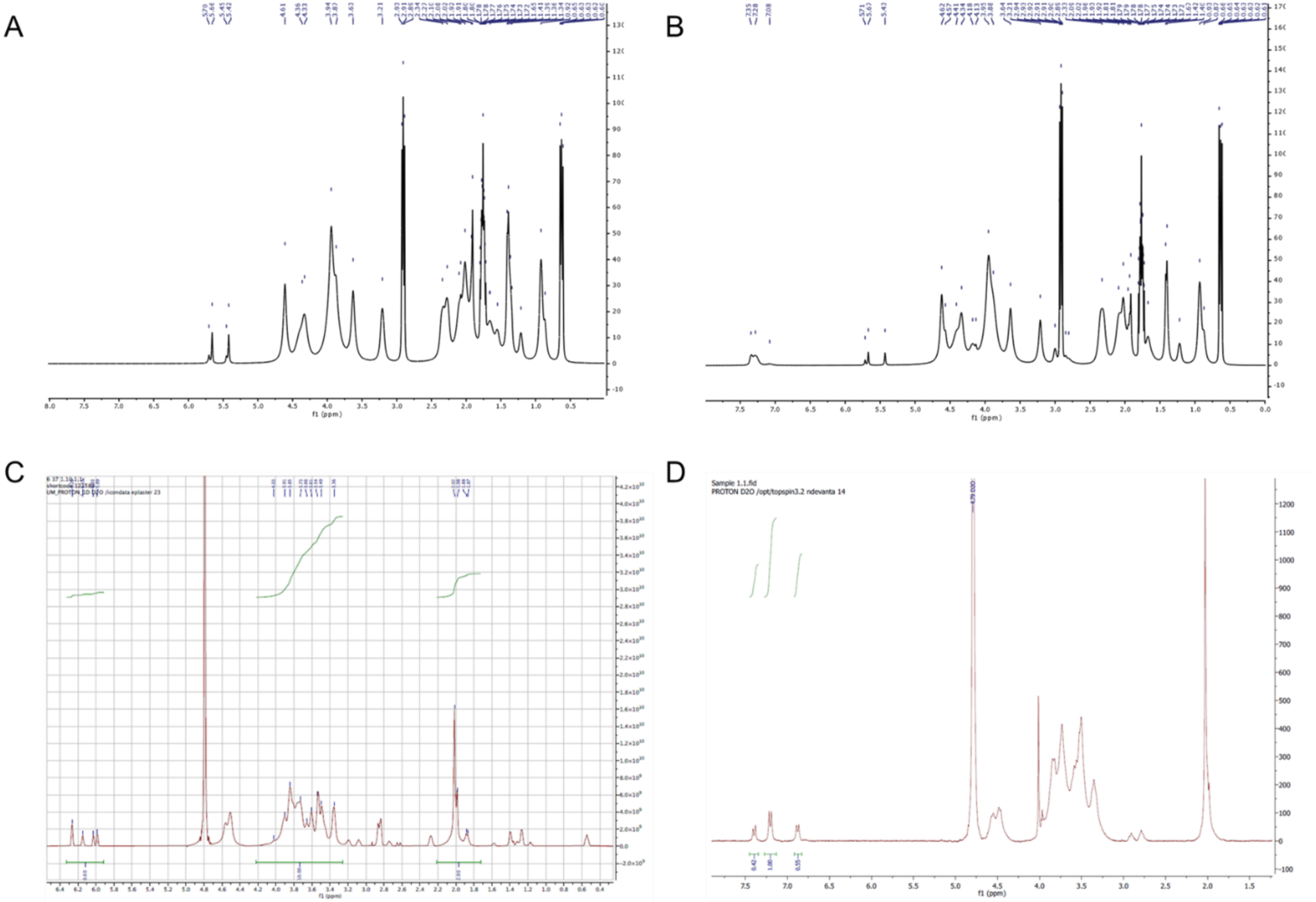

Supplementary figure S1: *^{1}H NMR spectrum of hydrogel precursors. (A, B) Gelatine methacryloyl (GelMA) in D_2O (4% concentration, with 0.5 mg/ml of 3-(Trimethylsilyl)-1-propanesulfonic acid sodium salt). NMR spectra of (A) GelMA50 and (B) GelMA80 confirm the methacrylation of the gelatine backbone. At 5.5 and 6.0 ppm the peaks*

corresponding to the protons belonging to the double bond of the methacrylate moiety with a shorter peak in GelMA50 compared to GelMA80. A small peak for lysine at 3.0 ppm was still present on the GelMA50 but it is missing on the GelMA80 sample. (C) Norbornene-functionalized hyaluronic acid (NorHA) in D_2O (5wt%). Modification of HA with norbornene (30%) determined by integration of vinyl protons (2x2H) relative to the sugar ring of HA (10H). NorHA precursor was the same tor preparing, norHA-DTT and norHA-MMP. (D) Tyramine functionalized hyaluronic acid (THA) in D_2O (3%w/v in D_2O containing 0.4 mg/ml hyaluronidase). The NMR spectrum conforms to the structure of the tyramine derivative of HA, showing a singlet corresponding to the N-acetyl group around 2ppm, and a broad multiplet between 3.2 and 4.0 ppm corresponding to various protons on the saccharide rings. The three peaks around 7 ppm corresponding to the aromatic resonances of the tyramine groups are visible (Loebel, Stauber et al. 2017). THA precursor was the same to prepare THA-collagen.

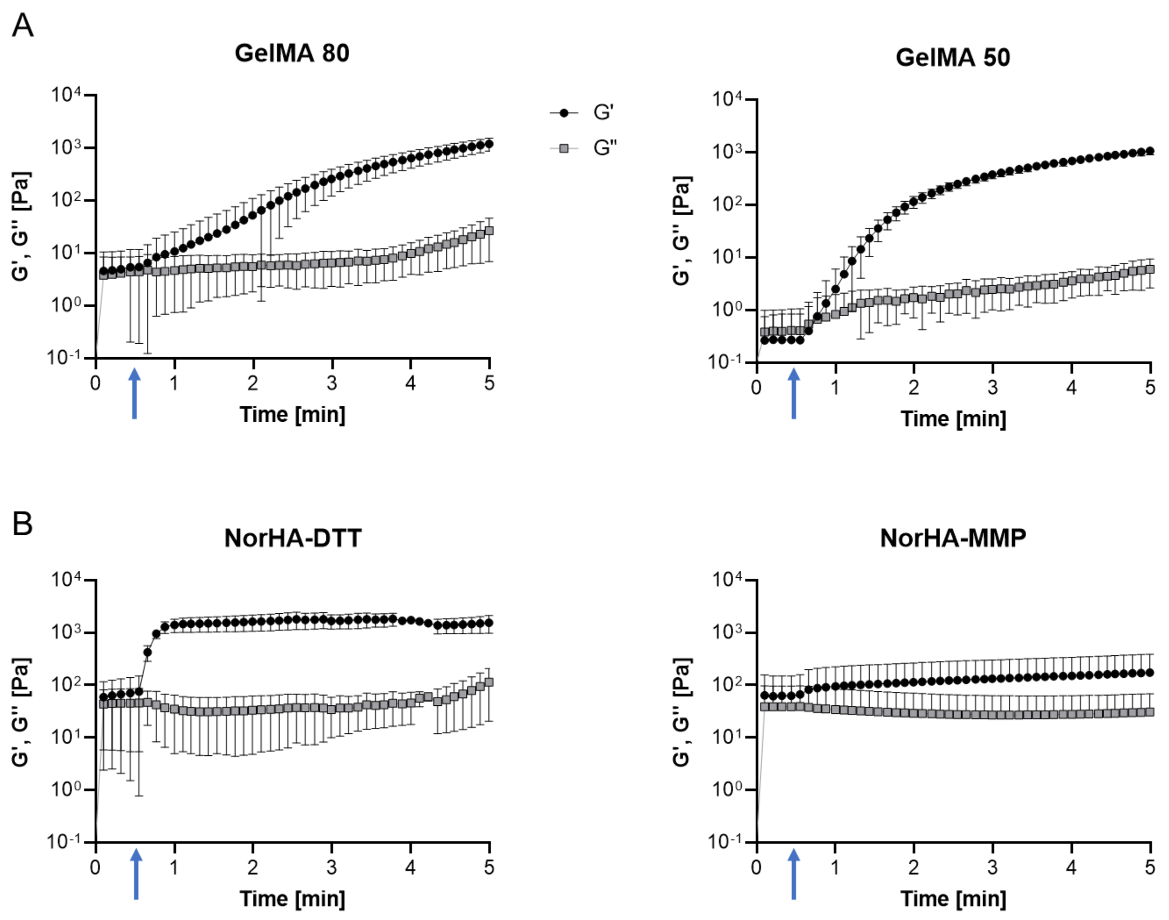

***Supplementary figure S2**: Photo rheology of gelatine methacryloyl (50% and 80% degree of functionalization, DOF) and norbornene functionalized hyaluronic acid (norHA) crosslinked with either a non-degradable (DTT) or MMP degradable crosslinker. Blue arrow indicates the time (30 sec) when the light was turned on for photo-crosslinking the hydrogel precursors.*

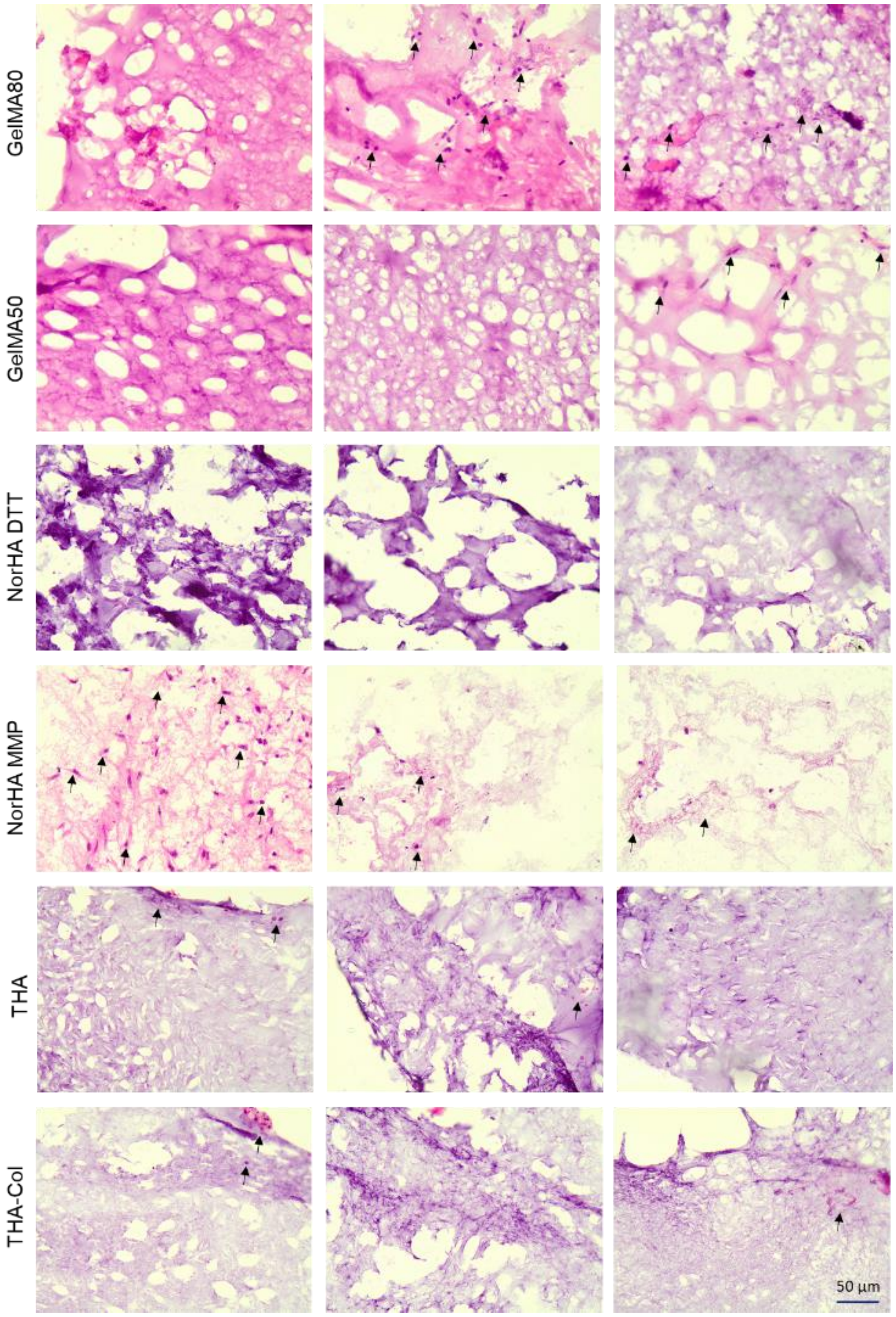

Supplementary figure S3*: Cell infiltration into hydrogels after 10 days implantation in semi-orthotopic model. HE staining of acellular hydrogels were polymerized in osteochondral defects and implanted in nude mice (n=3 samples per biomaterial). Most cells were observed in GelMA 80% and 50% as well as norHA MMP gels. Only single cells were present in the other hydrogel groups. Arrows indicate (multinucleated) cells. Scale bar 50 µm.*

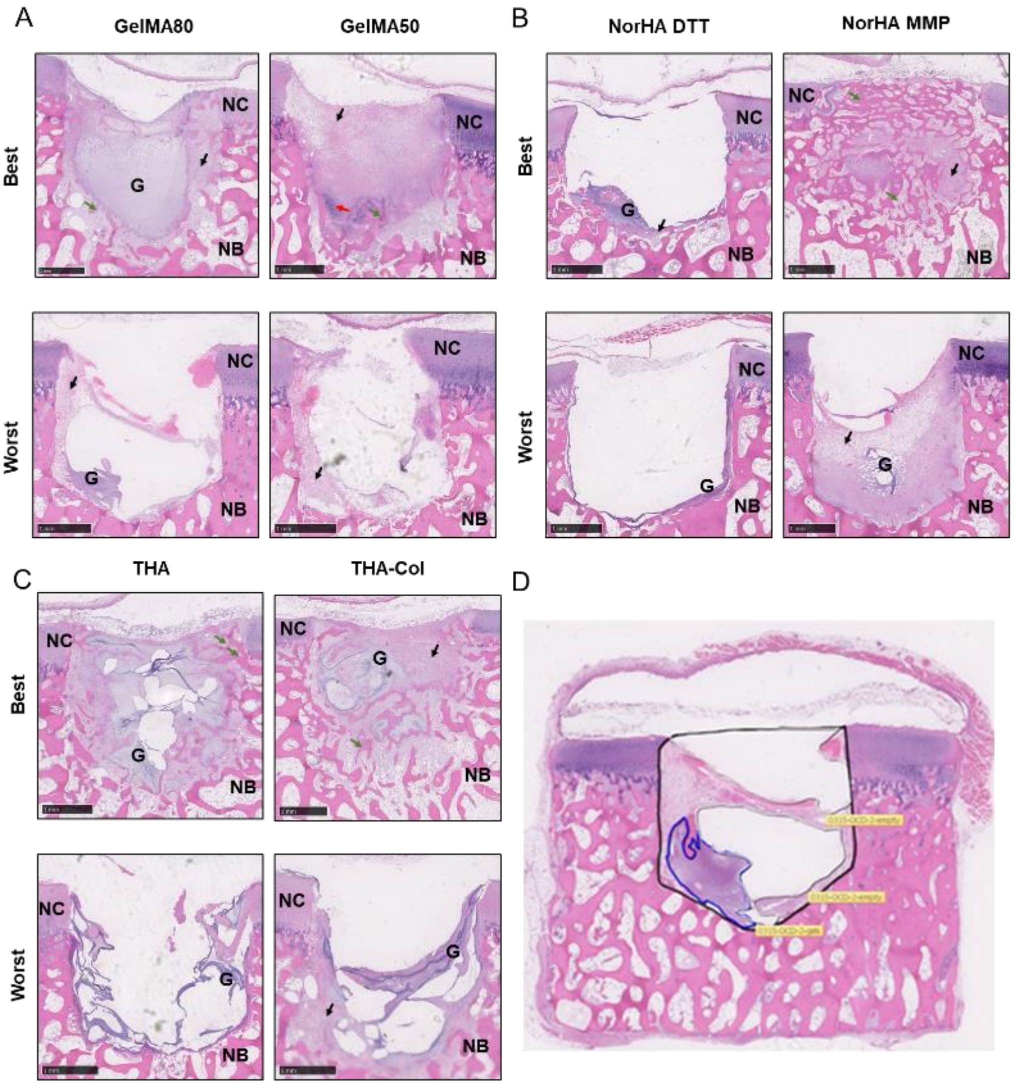

Supplementary figure S4*: Tissue regeneration in osteochondral defects filled with the hydrogels in an in vivo subcutaneous implantation mouse model. The best and worst repair constructs stained with H&E showing the in-situ hydrogels and tissue regeneration within the osteochondral defects after 6 weeks of implantation. A) GelMA degree of functionalization 50% and 80%, B) norHA with protease (MMP) cleavable and non-degradable DTT crosslinker, C) THA and THA-collagen. Scale bars indicate 1 mm. Black arrows indicated infiltrated cells within the defects. Red arrows indicated newly formed cartilage-like tissue. Green arrows indicated newly formed bone-like tissue. NC: native cartilage; NB: native bone; G: hydrogel. D) Example on defining the defect region (black line), hydrogel selection (blue line) and newly formed tissue formation (grey line) for quantification of tissue formation mice (n=5 samples per biomaterial).*

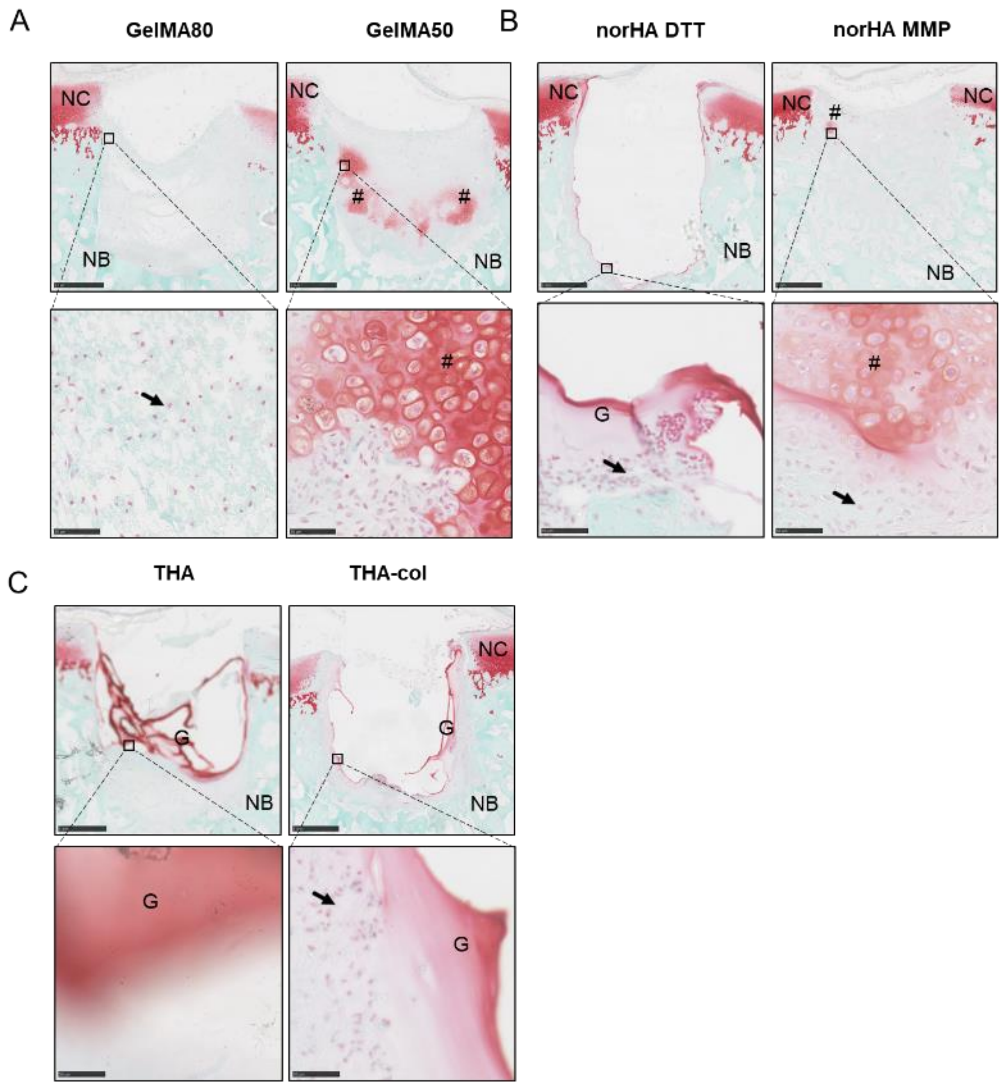

Supplementary figure S5*: Representative images of the repair constructs stained with Safranin O showing the in-situ hydrogels and tissue regeneration within the osteochondral defects after 6 weeks of implantation. A) GelMA degree of functionalization 50% and 80%, B) norHA with protease (MMP) cleavable and non-degradable DTT crosslinker, C) THA and THA-collagen. Scale bars indicate 1 mm (lower magnification) and 50 µm (higher magnification). Black squares indicated the magnified areas. Black arrows indicated infiltrated cells within the defects. NC: native cartilage; NB: native bone; G: hydrogel; #: cartilage-like tissue.*

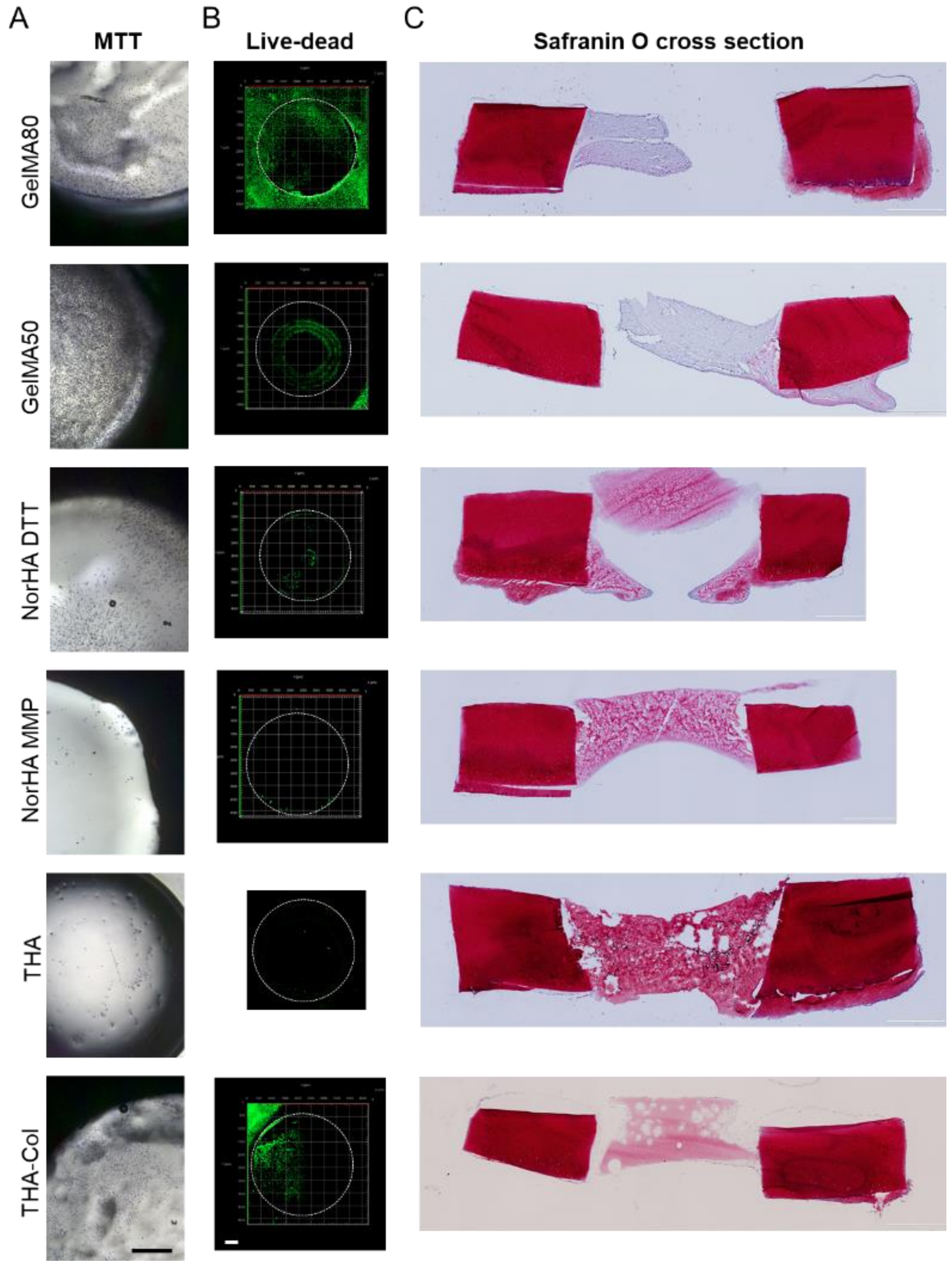

Supplementary figure S6: *Ex vivo cartilage explant model after 21 days of culture. A) Microscopic image of metabolic active chondrocytes migrating on the biomaterials (MTT staining). Scale bar 500 µm. B) Live-dead staining (green: alive cells, red: dead cells). Scale bar 500 µm. C) Safranin O staining of representative cartilage explants treated with biomaterials. Scale bar 1 mm. THA: tyramine modified hyaluronic acid (HA), THA-col, norHA DTT: norbonene functionalized HA with non-degradable crosslinker, norHA MMP: norHA with Matrix Metalloproteinase degradable crosslinker, GelMA 50: gelatine methacryloyl with 50% degree of functionalization (DoF), GelMA 80: GelMA 80% DoF.*
